# Production, Secretion and Biological Activity of *Bacillus cereus* Enterotoxins

**DOI:** 10.3390/toxins2071690

**Published:** 2010-06-29

**Authors:** Sonia Senesi, Emilia Ghelardi

**Affiliations:** 1 Dipartimento di Biologia,University of Pisa, via S. Zeno 37, 56127 Pisa, Italy; 2 Dipartimento di Patologia Sperimentale, Biotecnologie Mediche, Infettivologia ed Epidemiologia, University of Pisa, via S. Zeno 37, 56127 Pisa, Italy; Email: ghelardi@biomed.unipi.it

**Keywords:** hemolysin BL, non-hemolytic enterotoxin, cytotoxin K, *B. cereus*

## Abstract

*Bacillus cereus* behaves as an opportunistic pathogen frequently causing gastrointestinal diseases, and it is increasingly recognized to be responsible for severe local or systemic infections. Pathogenicity of *B. cereus* mainly relies on the secretion of a wide array of toxins and enzymes and also on the ability to undergo swarming differentiation in response to surface-sensing. In this report, the pathogenicity exerted by *B. cereus* toxins is described with particular attention to the regulatory mechanisms of production and secretion of HBL, Nhe and CytK enterotoxins.

## 1. Introduction

*Bacillus cereus* is a Gram-positive, spore-bearing rod that is widely distributed in the environment, namely soil, where spores persist under adverse conditions and can grow when readily decomposable matter is available. This motile bacterium is an aerobe or facultative anaerobe with large vegetative cells, typically organized in chains, ranging from 3–5 μm in length and 1 μm in width. *B. cereus* can grow over a wide temperature range (8–55 °C), but it is not well suited to tolerate low pH values (minimum 5–6) or water content (minimum water activity 0.95). Despite the fact that *B. cereus* can complete a full saprophytic life cycle, this bacterium may also behave as an opportunistic human pathogen. Long known to be responsible for two forms of food poisoning, characterized by either diarrhea and abdominal distress or nausea and vomiting, in recent years there has been an increasing concern over its potential to cause extra-intestinal infections in healthy individuals, but mostly in immunocompromised, critically ill, or otherwise debilitated patients. These infections include severe endophthalmitis, bacteremia, septicemia, endocarditis, pneumonia, meningitis, gastritis, and cutaneous infections [1,2,3]. 

*B. cereus* is the model species of the “*Bacillus cereus* group”, also known as *Bacillus cereus sensu lato*, comprising five other closely related species: *Bacillus anthracis*, *Bacillus thuringiensis*, *Bacillus mycoides*, *Bacillus pseudomycoides*, and *Bacillus weihenstephanensis* [1,4,5,6,7]. These bacteria share a significant degree of genetic similarity, so that both DNA-DNA hybridization [8], as well as 16S and 23S rRNA sequence analyses [9,10], have failed to clearly separate these taxa that are consequently considered as variants of a single species. Nevertheless, differences in both pathogenic properties and phenotypic traits of strains belonging to these subspecies have led to retention of their original nomenclature. In fact, while *B. anthracis* is the causative agent of the highly infectious disease anthrax [11], *B. cereus* is a soil bacterium behaving as a human opportunistic pathogen, and *B. thuringiensis* is an entomopathogenic bacterium used as a bio-pesticide worldwide [12]. *B. mycoides* and *B. pseudomycoides* [6], also classified as *B. mycoides* groups 1 and 2 [13], are environmental species that display rhizoidal growth on agar plates unlike other members of the *B. cereus* group. *B. weihenstephanensis* is distinguishable from mesophilic strains of *B. cereus* for its ability to grow aerobically at 7 °C but not at 43 °C and for the presence of typical signature sequences in the 16s rDNA and in the major cold shock gene *cspA* [5]. 

The production of plasmid-encoded virulence factors is responsible for the pathogenicity exerted by *B. anthracis* and *B. thuringiensis* in mammals and insects, respectively. Virulent *B. anthracis* strains are characterized by the presence of two large plasmids, pXO1 and pXO2, which carry genes coding for a tri-component protein exotoxin and a poly-d-glutamic acid capsule. The extra-chromosomal plasmid markers can be lost during strain propagation and naturally occurring strains without plasmids have been isolated from the environment. Conversely, other species of the group have been shown to occasionally contain these plasmids [14,15,16,17]. *Bacillus thuringiensis* is well known for its ability to produce parasporal crystalline protein inclusions (parasporal bodies or crystals) that are encoded by plasmids carrying *cry* genes [18]. Loss of these genes makes *B. thuringiensis* indistinguishable from *B. cereus* by other physiological or morphological traits. No virulence factors specific to *B. cereus* have been identified, and virulence proteins thought to be specific to *B. cereus* are present in all species of the *B. cereus* group, including *B. anthracis,* in which they are only weakly detectable [19]. It has also been shown that some *B. cereus* strains contain both rDNA operons with mesophilic signatures, and psychrotolerant signatures similar to *B. weihenstephanensis* [20], suggesting that intermediate forms between the two species might exist [21]. The broad occurrence of horizontal gene transfer in the *B. cereus* complex further complicates the taxonomy of this group of bacteria [22].

*B. cereus* is a motile bacterium harboring peritrichous flagella and most of the strains isolated from environmental, food, and clinical samples are able to swim in liquid media [23]. As for other flagellated bacteria, *B. cereus* flagella have been suggested to act as adhesins, thus favoring a stable interaction between the bacterium and epithelial cells [24]. 

While flagella are not essential for the production of biofilms by *B. cereus* [25], their presence and functionality are required for the collective movement of *B. cereus* across solid surfaces by swarming motility [26,27]. This specialized form of surface translocation is accomplished by a complex differentiation process of the swimmer cells, which respond to surface contact producing highly differentiated swarm cells [28]. These cells are longer, harbor a higher number of flagella, and acquire the ability to co-operatively move across various kinds of environmental surfaces, including mucosal surfaces. The wide distribution of this type of flagellum-dependent motility among *B. cereus* isolates [23], as well as in many other flagellated bacteria, suggests that swarming may be an advantage for the colonization of natural environments by this ubiquitous micro-organism. Moreover, swarming by *B. cereus* can also be influential in host-pathogen interactions, facilitating both host colonization and/or leading to an increase in the production of specific virulence factors. Indeed, differentiation of *B. cereus* swimmer cells into swarmers is associated with a significantly higher rate of secretion of the enterotoxin hemolysin BL (HBL) [23] and it also increases *B. cereus* pathogenicity in an experimental model of endophthalmitis in rabbits, independently of the secretion of virulence proteins [29]. This observation strongly suggests that swarming itself contributes to enhance the virulence potential this opportunistic pathogen may exert, by promoting a more rapid invasion of the host tissues by swarming-proficient *B. cereus* strains.

Due to its ability to form spores, *B. cereus* can survive a wide range of stress conditions such as those encountered in certain foods. *B. cereus* can be isolated from a wide variety of foods, including rice and pasta, milk and dairy products, infant foods, meat products, spices, fresh vegetables, seafood, and ready to eat foods. In addition, *B. cereus* represents one of the major pathogens in mass catering, because its elimination is not guaranteed by pasteurization and sanitation procedures [30]. 

Food poisoning by *B. cereus* can either be caused by an infection or an intoxication, which leads to a diarrheal or an emetic type of illness, respectively. Foods often related to diarrheal food poisoning include meat products, soups, vegetables, sauces and dairy products, while those related to the emetic type of syndrome are mainly rice and pasta [31]. The diarrheal type of illness is thought to be caused by the production of enterotoxins by *B. cereus* in the human small intestine after consumption of contaminated food [31]. The emetic type of illness is caused by production of the emetic toxin cereulide by *B. cereus* in foods before consumption and causes nausea and vomiting [30]. As these toxins can be produced by all the other members of the *B. cereus* group, particularly *B. thuringiensis*, *B. mycoides* and B. *weihenstephanensis*, food poisoning can even be caused by these different species [32]. Generally, symptoms caused by *B. cereus* food poisoning are regarded as mild, and therefore *B. cereus* food poisoning is probably under reported.

Among the enterotoxins produced by *B. cereus*, the hemolytic enterotoxin hemolysin BL (HBL), the non-hemolytic enterotoxin (Nhe), and cytotoxin K (CytK) are claimed to play a major role in diarrheal disease and will be addressed in this review. HBL andNhe are tripartite toxins [33,34], while CytK is a hemolytic toxin with homology to the *β*-barrel pore-forming toxins [35,36]. The potential diarrheal effect these toxins exert is believed to rely on their ability to act as tissue-destructive/reactive proteins damaging the integrity of the plasma membrane of several cells, epithelial cells of the small intestine included. However, with the exception of HBL (Section 2), a direct involvement of these toxins in diarrhea has not been clarified by the use of suitable animal models. In addition, as *B. cereus* strains causing the diarrheal disease generally produce a variety of other toxins and enzymes, the exact role of these toxins in the pathogenesis of this illness is still unknown.

## 2. Hemolysin BL

### 2.1. Structure and Biological Activity

Hemolysin BL (HBL) is a membrane-lytic system composed of three antigenically distinct proteins designated B, L_1_, and L_2_[33]. These proteins are secreted independently and all three are necessary for maximal biological activity [34,37]. To date, the toxic activities identified for HBL include hemolysis, vascular permeability and necrosis in rabbit skin [33,38], *in vitro* degradation of explantedrabbit retinal tissue, and *in vivo* ocular necrosis and inflammationin rabbits [39]. In regard to HBL diarrheal potential, this toxin has been shown to cause rapid fluid accumulation in ligated rabbit ilealloops [37], which is a decisive test to reveal the diarrheal activity of enterotoxins [40]. In this assay, the potency of HBL was in a range approaching the potency of cholera toxin, thus suggesting that it could be considered a primary virulence factor in *B. cereus* diarrhea [37]. 

HBL is secreted by about 45–65%of *B. cereus* strains [41,42]; however, variations in the percentage of HBL-producing strains have been reported among clinical (81%) and environmental/food isolates (43%) [23]. 

**Figure 1:**
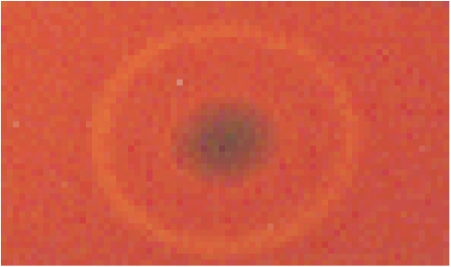
Discontinuous pattern of hemolysis produced by HBL on sheep blood agar.

A distinctive feature of HBL is an unusual discontinuous hemolysis pattern produced in blood agar [33,43]. When HBL diffuses from a bacterial colony or a well in blood agar, lysis begins away from the well or colony, followed by slow lysis nearer the source (Figure 1). 

It has been proposed that the three HBL proteins bind to erythrocytes independently and the membrane-associated HBL components form a membrane attack complex that causes lysis by a colloid osmotic mechanism [43]. This paradoxical zone phenomenon has been explained with a model in which a cell will not undergo lysis unless it has been primed with the B component, whose binding to the cell is inhibited by excess L_1_. Near the source, colony or well, L_1 _diffuses rapidly and accumulates to inhibitory levels before cells are primed. Hemolysis begins in a zone in which the B concentration is high enough to prime cells that are then rapidly lysed by any concentration of L_1+2_ [43].

**Figure 2:**
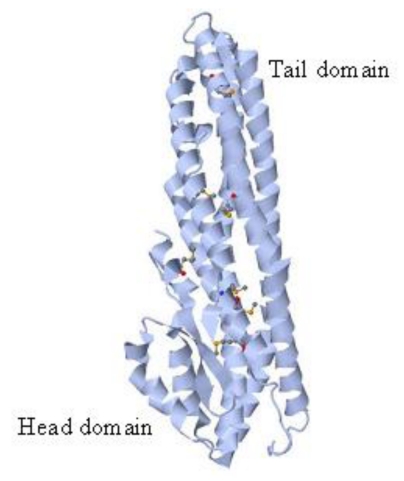
X-ray crystal structure of the B component of HBL (Protein Data Bank; http://www.pdb.org/). The α/β head domain has been proposed to bind and insert into the membrane of susceptible cells [44].

The X-ray crystal structure of the B component of HBL, deposited in the Protein Data Bank (PDB ID: 2NRJ), reveals a long α-helical bundle and a small α/β head domain [44] (Figure 2). 

This structure is highly similar to that of *E. coli* hemolysin E (HlyE, ClyA, SheA) (PDB ID: 1QOY) [45] despite the low sequence homology, suggesting a common mode of pore formation. Based on the structural homology of the HBL-B component and HlyE and on analytical gel filtration studies, a model of HBL pore formation has also been proposed in which oligomerization of the B component into a heptamer or octamer may form a pore [44]. In this model, the requirement of the three HBL components for hemolysis was explained by suggesting a role for both L_1_ and L_2_ in inducing conformational changes in B or in stabilizing the head domain of this protein in a membrane-insertion/competent conformation [44].

### 2.2. Genes and Regulation of Gene Transcription

The HBL proteins B, L_1 _and L_2_ are encoded by *hblA*, *hblD*, and *hblC*, respectively [46]. These genes are arranged in an operon in the transcriptional order *hblC*, *hblD*, and *hblA* [46]. Immediatelydownstream from *hblA*, lies an open reading frame, *hblB*, which isabout 85% identical to *hblA* in the first 158 predicted aminoacids. The *hblB* gene product has not yet been isolated. In the prototype strain F837/76, the first in which HBL has been characterized; B, L_1 _and L_2_ have molecular weights of 37.5, 38.2, and 43.5 kDa, respectively. However, many isolates produce more than oneantigen reactive to antibodies against individual HBL components [47] and distincthomologous variants of each protein from a single *B. cereus* strain was also shown [48]. The high degree of similarity among these homologues suggests that the HBL genes may have been subjected to either simultaneous duplication or horizontal transfer [48].

Expression of *hbl* genes has been shown to be regulated by the proteins PlcR [49], ResD [50,51], Fnr [52,53,54], and CcpA [55]. PlcR is known to autoactivate and activate the expression of the *hbl* operon by binding to a specific sequence, the PlcR-box palindromic region (TATGNAN4TNCATA) in the operon promoter sequence [49,56]. The transcription of *plcR* starts at the onset of the stationary phase and is repressed by the sporulation factor Spo0A [57]. PlcR is activated by PapR, an autoinducer peptide that accumulates inside bacteria when high cell densities are reached, and facilitates binding of PlcR to the PlcR box [58]. ResD is the response regulator of the two components system ResDE, which is activated under low-oxidoreduction potential anaerobic conditions [50]. ResD regulates the expression *hbl* genes directly interacting with the promoter regions of the *hbl* operon as well as the enterotoxin regulator genes *plcR*, *resDE*, and *fnr* [51]. The redox regulator Fnr is required for full expression of *hbl* genes independent of oxygen tension [52] and carbohydrate used as a carbon source [54]. This protein activates the expression of *hbl* genes by binding to the promoter sequences *phbl* and *pplcR* [53]. The catabolite control protein CcpA is a transcriptional regulator that binds to DNA at a specific cis-binding sequence, the Catabolite Responsive Element (CRE) [55]. In the stationary phase of growth and in the presence of glucose, this protein is thought to repress the expression of *hbl* genes, by binding to putative CRE-sites identified in this operon [55]. Therefore, HBL production by *B. cereus* is highly controlled, as a wide variety of signals and proteins act as regulators of *hbl* gene transcription. 

### 2.3. Secretion

Analysis of the amino acid sequences of the three HBL proteins show that they all possess a signal peptide sequence at their amino termini, thus suggesting their secretion through an *S*-dependent secretion pathway (Sec system). Nevertheless, integrity of the flagellar export apparatus has been demonstrated to be required for secretion of HBL [23,59]. The flagellar export apparatus encompasses at least six integral proteins (FlhA, FlhB, FliO, FliP, FliQ, and FliR in *Salmonella*) [60] that share substantial homology with components of the type III virulence secretion pathway found in Gram-negative bacteria [61]. This apparatus is essential for the export of flagellar components that are sequentially assembled from the cytoplasmic membrane outward. 

The first demonstration that the flagellar export complex could also function for secretion of non-flagellar proteins came from studies on *Yersinia enterocolitica*, in which the virulence-associated protein YplA is secreted by the flagellar export apparatus [62]. This finding, combined with the structural similarity of this export system with the type III secretion system, have suggested that they may have had a common evolutionary origin and share overlapping functions. Regarding HBL, this protein was shown to be retained and degraded inside the cytoplasm in an acrystalliferous *B. thuringiensis* mutant in *flhA* [59]. Failure to secrete intracellularly-synthesized HBL was also demonstrated in *B. cereus* natural isolates lacking flagella [23]. A significant reduction in the amount of secreted HBL was reported for a *B. cereus* mutant lacking FlhF, a signal recognition particle (SRP)‑like GTPase involved in the regulation of the number and arrangement of flagella on the bacterial surface [27]. This mutant was characterized by a marked reduction in the number of flagella (1–3 per cell) in comparison to wild-type (10–12 per cell) [27]. Conversely, hyperflagellated swarm cells of *B. cereus* secrete significantly higher levels of HBL than the vegetative swimmer cells [23]. All these data stress the role played by flagella in the virulence exerted by infectious *B. thuringiensis/B. cereus* strains; indeed, these locomotion organelles may facilitate adhesion to host cells, contribute to increase HBL secretion, and confer a higher propensity to colonize host mucosal surfaces and further enhance the flagellum‑mediated HBL secretion through differentiation of hyperflagellated swarm cells.

Although further studies are still needed to clarify the mechanism of HBL secretion, the finding that the molecular mass of the secreted HBL components corresponds to that estimated for the proteins lacking the amino-terminal signal sequence, suggests that signal peptidases cooperate with the flagellar export apparatus for toxin secretion. This hypothesis is supported by the observation that the flagellar P- and L-ring subunits, which do possess a signal peptide sequence before being secreted, are exported and assembled in the flagellar structure only when all the components of the flagellar export apparatus are active [63].

## 3. The Non-Hemolytic Enterotoxin Nhe

### 3.1. Structure and Biological Activity

The three-component enterotoxin Nhe was first isolated from a *B. cereus* strain involved in alarge food-poisoning outbreak in Norway [64]. The proteins were different from the components of HBL and had no evident hemolytic activity. It was initiallysuggested that a collagenase of 105 kDa [65] was part of the Nhe complex [64], but this was later found to be incorrect. Sequencingof the operon encoding the two components NheA and NheB [66] revealeda novel gene encoding the protein designated NheC subsequently shown to be part of Nhe [34]. 

Nhe isproduced by almost 100% of *B. cereus* strains [42]. Nhe is cytotoxic against Vero cells [34] and the cytotoxicity of supernatants from strains unable to synthesize HBL can be abolished by the addition of a neutralizing monoclonal antibody directed against NheB [67]. As described for HBL, Nhe proteins are secreted independently and maximal toxic activity on Vero cells requires all three components in a molar ratio 10:10:1 of NheA, NheB, and NheC, respectively [34]. NheB is the binding component of the enterotoxin complex and an increase in the concentration ofNheC results in a decrease in Nhe toxic activity [34].

The cytotoxic activity of Nhe on epithelial cells has been shown to be due to colloid osmotic lysis following pore formation in the plasma membrane [68]. The same study also demonstrated hemolytic activity of Nhe, although lower than HBL, and homology models showed common structural features shared by the B component of HBL [44] and the B and C components of Nhe [68]. 

### 3.2. Genes and Regulation of Gene Transcription

The three Nhe proteins are encoded by the *nhe* operon, comprising *nheA*, *nheB*, and *nheC* [34,66]. Primer extension analysis of the *nhe* operon identified the transcriptional start site at a Tpositioned 62 and 66 base pairs upstream of the translational startsite in two *B. cereus* strains, respectively [34]. The presence of an inverted repeat between *nheB* and *nheC* suggested that translational repression could act in regulating the *nheC* expression, *nheC* being expressed at a lower level than *nheA* and *nheB* [34,66].

The same transcriptional regulators described above for HBL are also active in modulating the expression of *nhe* genes. A PlcR-box present in the promoter region of the *nhe* operon is responsible for the PlcR-dependent activation of *nhe* transcription [49,56]. ResD and Fnr directly interact with the promoter region of the structural operon *nhe* activating its expression [51,52] and CcpA repress the expression of this operon by binding to putative CRE-sites [55]. 

Despite the fact that regulators of *nhe* and *hbl* gene expression appear to be identical, the analysis of mutants defective in *fnr* and *ccpA* demonstrated different effects on the expression/secretion of these toxins. In fact, Nhe secretion was shown to be more moderately affected than HBL secretion in the *fnr* mutant of *B. cereus* [54]. A *ccpA* deletion mutant displayed a higher expression (almost 20-fold) of the *nhe* operon compared to the *hbl* operon in the stationary phase [55].

### 3.3. Secretion

The presence of signal peptide sequences in the three components of Nhe suggests their secretion occurs through the Sec system, although no direct demonstration of a Sec-dependent secretion of Nhe has been provided. The only data regarding Nhe secretion in *B. cereus* came from the observation that toxin secretion is influenced by FlhF [27], a signal recognition particle-like protein showing homology with Ffh and FtsY that are required for extracellular accumulation of proteins in *E. coli* and *B. subtilis* [69,70]. The *B. cereus* mutant in *flhF* was shown to display an almost 20% increase in the secretion of Nhe, together with a general increase in secretion for almost all extracellular proteins [27]. Conversely, the *B. cereus* mutant in *flhF*, which is characterized by a reduction in the number of flagella on the cell surface, displays a significant decrease in the secretion of HBL, whose export is flagellum-dependent. Therefore, it appears that Nhe secretion does not require a flagellar export apparatus to be released outside the cell and, though only indirectly, supports the hypothesis that Nhe secretion can occur *via* a Sec-dependent mechanism. 

## 4. Cytotoxin K

CytK was first identified as the only cytotoxic, necrotic, and hemolytic toxin of the *B. cereus* strain NVH391/98, which was involved in a severe outbreak of food poisoning occurring in France in 1998 [36]. In the same year, this 34 kDa protein was also identified from fractionated supernatants of an endophthalmitis-associated *B. cereus* isolate and referred to as hemolysin IV [39]. CytK was purified and proved to be highly cytotoxic against Vero cells [35]. The deduced amino acid sequence of CytK shows similarity to *Staphylococcus aureus* leucocidins, gamma-haemolysin and alpha-haemolysin, *Clostridium perfringens* beta-toxin and *B. cereus* haemolysin II, all belonging to a family of beta-barrel channel-forming toxins [36]. In agreement with this structural similarity, CytK was demonstrated to possess enterotoxic activity against intestinal epithelia and an ability to form pores in planar lipid bilayers [35]. 

Cytotoxin K can occur in two different forms, CytK 1 and CytK 2. CytK 1 was originally isolated from strain NVH391/98 and was observed to be five-fold more cytotoxic for human intestinal CaCo2 cells and Vero cells than CytK 2 [71]. However, the finding that other strains carrying CytK 1 are not as toxic as NVH391/98 [72] suggests that variations in cytotoxicity are actually linked to variations in the levels of toxin expression instead of different virulence potential of the two CytK forms. 

CytK is encoded by *cytK*, whose expression is regulated by PlcR through its binding to a PlcR-box in the cytK promoter sequence [36,56]. No putative CRE-site was identified upstream of *cytK* and differential expression of this gene was not observed in a *ccpA* mutant, suggesting that *cytK* expression is not CcpA-mediated [55]. 

*cytK* was recently detected in about 85% of *B. cereus* and *B. thuringiensis* strains belonging to a collection of 616 strains [73], indicating the high prevalence of strains potentially able to cause severe food poisoning.

The aminoterminal sequence of CytK contains a putative secretory signal peptide suggesting its secretion through the Sec-pathway. CytK secretion was shown to be unaffected in an acrystalliferous *B. thuringiensis* mutant in *flhA* lacking flagella [59], thus indicating that the flagellar export system is not involved in the secretion of this toxin. 

## 5. Conclusions

The production of spores, which are highly adhesive and can spread from natural *B. cereus* habitats to food production environments, accounts for the ability of *B. cereus* to contaminate any kind of food. The high frequency of food contamination by *B. cereus* and the active production and secretion of HBL, Nhe and CytK enterotoxins, explain why this organism is responsible for the majority of food-poisoning related diseases. However, increasing evidence supporting the involvement of *B. cereus* in severe extra-intestinal infections highlights the importance to further study: (i) the mechanisms by which the toxins and enzymes *B. cereus* produces contribute to development and progression of diseases; and (ii) the role played by swarming migration in enhancing tissue colonization by infectious *B. cereus* strains.
